# High Sensitivity of Human Adipose Stem Cells to Differentiate into Myofibroblasts in the Presence of *C. aspersa* Egg Extract

**DOI:** 10.1155/2017/9142493

**Published:** 2017-12-27

**Authors:** Natalio García-Honduvilla, Alberto Cifuentes, Miguel A. Ortega, Arancha Delgado, Salvador González, Julia Bujan, Melchor Alvarez-Mon

**Affiliations:** ^1^Department of Medicine and Medical Specialties, Faculty of Medicine and Health Sciences, University of Alcalá, Alcalá de Henares, Madrid, Spain; ^2^Networking Biomedical Research Center on Bioengineering, Biomaterials and Nanomedicine (CIBER-BBN), Alcalá de Henares, Madrid, Spain; ^3^Ramón y Cajal Institute of Sanitary Research (IRYCIS), Madrid, Spain; ^4^University Center of Defense of Madrid (CUD-ACD), Madrid, Spain; ^5^Industrial Pharmacy Cantabria, Madrid, Spain; ^6^Immune System Diseases-Rheumatology and Oncology Service, University Hospital Príncipe de Asturias, Alcalá de Henares, Madrid, Spain

## Abstract

**Introduction:**

Regeneration therapy using adipose-derived stem cells (ADSC) has been proposed in the treatment of skin aging. Myofibroblast plays a relevant role in the organization of the extracellular matrix of the damaged skin. A natural extract was derived from the eggs of the mollusk *Cryptomphalus aspersa* (e-CAF) that seems to play a role on skin repair. We have investigated the potential effects of e-CAF in the differentiation of ADSC.

**Materials and methods:**

ADSC were cultured in the absence or presence of e-CAF (50 and 200 *μ*g/mL) for 24 hours and 7 days. Real-time cell assay, morphological, immunofluorescence, and RT-PCR techniques were used to evaluate the cell culture and expression of *α*SMA, collagen I, and tropoelastin.

**Results:**

e-CAF induced significant reduction in the rate of growth of ADSC from 24 hours to 7 days of culture. e-CAF also induced bigger sizes, higher levels of cytoplasmic refringence and complexity, and a more polyhedral morphological changes in the cultured ADSC. The protein and mRNA expression of *α*SMA was significantly increased in e-CAF-cultured ADSC.

**Conclusion:**

e-CAF promotes ADSC differentiation to myofibroblasts and should be considered as a potential agent for the prevention and treatment of skin aging.

## 1. Introduction

Aging is a complex physiological process which causes a progressive decrease in the functionality of all human tissues, including skin. In the course of aging, the skin is subject to a number of agents or conditions which, together, are known as exposome. The skin exposome consists of external and internal factors and their interactions, as well as the response of the human body to these factors that lead to biological and clinical signs of skin aging [1]. Among the internal factors, genetics, metabolism, and hormones should be highlighted, while the main environmental factors include solar radiation and pollution, which add up to trigger photoaging [2, 3].

The clinical signs of aged skin manifest as wrinkles, dermal atrophy, and impaired wound healing, in part due to alterations in the organization and remodeling process of the extracellular matrix (ECM). Its main components, collagen and elastin, provide strength and elasticity to the tissue. Their degradation, which is increased during aging, elicits loss of structural integrity and decreased repair ability [2, 4].

After the skin is injured, repair mechanisms are activated, including transforming growth factor-beta (TGF*β*) expression, which induces fibroblast proliferation and migration to the wound site, and their differentiation towards myofibroblasts by increasing the expression of *α*-smooth muscle actin protein (*α*-SMA), promoting the secretion and assembly of new ECM components that renew the skin structural support and being responsible for tissue contractility during maturation [5, 6]. During aging, the loss of ECM compromises this process and impairs the competence of the skin to successfully ensure scar formation and wound healing [7–9].

New treatments approaching the endogenous regenerative properties of the skin have been under extensive research in the last years [10], being especially promising are those focused in the fields of tissue engineering and regenerative medicine. Adipose-derived stem cells (ADSC) have emerged as a particularly relevant cell population to be targeted for these purposes. Their abundance, accessibility, and minimally traumatic harvesting, along with their ability to differentiate into several cell lineages, make ADSC a great candidate for advanced therapeutic uses [11–14]. ADSC can act in a paracrine fashion by secreting an assortment of growth factors, including insulin-like growth factor (IGF), vascular endothelial growth factor (VEGF), transforming growth factor-beta 1 (TGF-*β*1), and hepatocyte growth factor (HGF) [15, 16]. Besides, in addition to the potential of these cells to differentiate to osteogenic, chondrogenic, and adipogenic lineages, several studies have shown their capability to transform into cells involved in cutaneous repair, such as endothelial cells, keratinocytes, fibroblasts, and myofibroblasts [15–19]. ADSC have wound-healing and antioxidant effects on human skin via secretion of growth factors and activation of dermal fibroblasts. Thus, ADSC and its secretory factors are effective in wrinkles resulting from photoaging. The antiwrinkle effect is mainly mediated by reducing UVB-induced apoptosis and stimulating collagen synthesis of human dermal fibroblasts [12–14]. However, it has been described that age can cause changes in the function and differentiation ability of ADSC [20]. Therefore, the use of agents able to attenuate these effects could be useful in the treatment of skin aging.

In this regard, natural ingredients derived from the mollusk *Cryptomphalus aspersa* have been developed and assessed as regenerative agents for cutaneous tissue [16–20]. Its secretion (SCA) has been demonstrated to possess antioxidant and skin regenerative properties, preserving the survival of keratinocytes and fibroblasts and ECM dynamics, while promoting their mitogenic and motogenic activities during wound healing [21–27]. Moreover, a novel ingredient derived from an extract of the eggs of *C. aspersa* (e-CAF) has been recently developed, showing promising effects for the treatment of skin aging. In a recent study using human keratinocytes (HaCaT), human dermal fibroblasts (HDF), and senescent fibroblasts (SHDF), it has been demonstrated that e-CAF is able to induce migration and ECM production in these cell lines, while improving cytoskeletal organization. Furthermore, e-CAF showed antiaging properties, reducing the expression of age-related markers and preventing cell death due to ultraviolet radiation [28]. Furthermore, e-CAF has shown to promote migration of human hair dermal papilla cells (HDDPCs), also increasing their migratory behaviour and modulating the expression of adhesion molecules, suggesting its possible role on skin regeneration and prevention of tissue damage and skin aging [29].

In the present work, the in vitro effects of e-CAF on human ADSC have been assessed. Firstly, the cell proliferation and morphology of the natural extract derived from the eggs of mollusk *Cryptomphalus aspersa*, e-CAF, was assayed. For that, real-time cell assay (RTCA) was used to assay the capacity of e-CAF to modulating ADSC growth and size from 24 hours to 7 days of cultures. Likewise, the extract showed signs of a prodifferentiation activity. Subsequent analysis of myofibroblast markers showed an increase of this cell type in treated ADSC, pointing to an inductive effect of e-CAF towards this cell lineage. For this determination, the selected markers were expression of *α*SMA, a cytoskeletal protein commonly used as a myofibroblast marker. These data suggest that e-CAF might modulate the effect in the ADSC differentiation. This activity could be considered as a potential photoaging agent due to capacity of tissue repair and prevention of aging.

## 2. Materials and Methods

### 2.1. Cell Culture

Human ADSC were purchased from a commercial supplier (StemPro®, Gibco®, Thermo Fisher Scientific, Waltham, MA, USA) and cultured in MesenPro RS™ medium (Gibco) in a humidified incubator at 37°C in a 5% CO_2_ atmosphere. The cells were maintained at subconfluence and were subcultured when necessary. All experiments were carried out using cells from the second to the fourth passages.

### 2.2. Ingredient

e-CAF (Industrial Farmacéutica Cantabria, S.A., Madrid, Spain), an ingredient derived from the eggs of *C. aspersa*, was prepared as described by Espada et al. [28]. Briefly, the spawn was collected, rinsed at low pressure, immersed in saline solution, and kept at between 2°C and 8°C. Then, intact snail spawn was obtained by filtration through a mesh and lysed. e-CAF was homogenized in DMEM (Gibco) and filtered through 0.22 *μ*m filters to create a stock solution, and total protein concentration was measured by Bradford, as elsewhere described. Treatment media were prepared by mixing 4/5 of MesenPro RS with 1/5 of the stock solution and/or basal DMEM, to a final concentration of 50 *μ*g/mL (e-CAF-50 group), 200 *μ*g/mL (e-CAF-200 group), and 0 *μ*g/mL (CTRL group). Cells belonging to each of the study groups were cultured in the corresponding medium for 7 days, receiving a cell culture medium change at day 4.

### 2.3. Real-Time Cell Assay (RTCA) and Morphological Studies

The growth of the different groups was measured using a label-free, impedance-based RTCA system (xCELLigence RTCA SP, Roche Diagnostics, Basel, Switzerland). ADSC were seeded in 96-well E-plates at 2000 and 1000 cells per well and maintained in growth medium for 24 hours, before switching to the different treatment media. Subsequently, cell growth was studied for 7 days, before the cultures could reach confluence. Cell index (CI) was recorded every 30 minutes and normalized to the values at 24 h. For each group and cell concentration, the mean slope of the curves between the start of the treatment and the end of the experiment was calculated. Duplicate parallel plates were prepared in regular clear 96-well plates to allow the visualization and photography of the cultures during the experiment, using an Axiovert 40C inverted microscope equipped with an AxioCam ICc1 digital camera (Carl Zeiss, Oberkochen, Germany). Three independent experiments were carried out, each of them in triplicates.

To further assess the morphology and size of the cells, ADSC were seeded onto sterile 12 mm diameter glass coverslips and cultured as described above. Cells were fixed with 4% paraformaldehyde, subjected to standard haematoxylin-eosin staining and visualized in a Zeiss Axiophot microscope equipped with an AxioCam HRc digital camera (Carl Zeiss). The mean area of the cells in each group of three different experiments was measured in nine random high-magnification fields (10 cells/field) using ImageJ software (NIH, Bethesda, MD, USA).

### 2.4. Immunofluorescence

The presence of *α*SMA, a myofibroblast marker, was detected and quantified by immunofluorescent labelling. Fixed cells were permeabilized with 0.1% Triton X-100, blocked with 3% bovine serum albumin, and incubated with anti-*α*SMA monoclonal antibody (clone 1A4, Sigma-Aldrich, St. Louis, MO, USA). FITC-labelled anti-mouse antibody (Sigma-Aldrich) was used as secondary antibody, and nuclei were counterstained with DAPI as elsewhere described. Negative controls, exposed to 3% BSA instead of primary antibody, were included. Cells were observed using a Leica TCS SP5 confocal microscope (Leica Microsystems, Wetzlar, Germany), and the percentage of *α*SMA-positive cells of three independent experiments was evaluated in nine random high-magnification fields.

### 2.5. Real-Time PCR

Total RNA was isolated from the cultures by standard guanidinium thiocyanate-phenol-chloroform extraction procedures using TRIzol (Thermo Fisher Scientific). RNA was recovered from the aqueous phase by precipitation, and its quantity and purity were assessed by measuring optical density at 260/280 and 260/230 nm in a NanoDrop ND-1000 spectrophotometer (Thermo Fisher Scientific).

Complementary DNA was synthesized from 200 ng of total RNA by reverse transcription (RT) with oligo-dT primers and the M-MLV reverse transcriptase enzyme (Thermo Fisher Scientific). cDNA was quantified by real-time PCR, using the relative standard curve method, in a StepOnePlus™ System (Thermo Fisher Scientific). The following primers were used: *α*SMA (Fwd 5′-AGC GTG GCT ATT CCT TCG TT-3′ and Rev 5′-CCC ATC AGG CAA CTC GTA ACT-3′), collagen type I (Fwd 5′-CCA TGT GAA ATT GTC TCC CA-3′ and Rev 5′-GGG GCA AGA CAG TGA TTG AA-3′), tropoelastin (Fwd 5′-GTG TAT ACC CAG GTG GCG TG-3′ and Rev 5′-CGA ACT TTG CTG CTT TAG-3′), and GAPDH (Fwd 5′-GGA AGG TGA AGG TCG GAG TCA-3′ and Rev 5′-GTC ATT GAT GGC AAC AAT ATC CAC T-3′). The samples were subjected to an initial stage of 10 min at 95°C, followed by 40 cycles of 15 s at 95°C, 30 s at 59.5°C (TE), 59.9°C (*α*SMA) or 60.0°C (Col-I, GAPDH), and 1 min at 72°C. Four independent experiments were carried out, and each experiment was run in triplicates, including a no-template control in each reaction. GAPDH was used as reference gene.

### 2.6. Statistical Analysis

Statistical software GraphPad Prism 5 (GraphPad Software Inc., La Jolla, CA, USA) was used to analyse the data. Groups were compared using ANOVA followed by Tukey-Kramer test. Non-Gaussian data sets (RT-PCR) were normalized by logarithmic transformation prior to statistical testing. Data are expressed as mean ± standard error of the mean (SEM). Levels of significance were set at ^∗^*p* ≤ 0.05, ^∗∗^*p* ≤ 0.01, and ^∗∗∗^*p* ≤ 0.001.

## 3. Results

### 3.1. e-CAF Modulates ADSC Growth and Size

**Figure 1:**
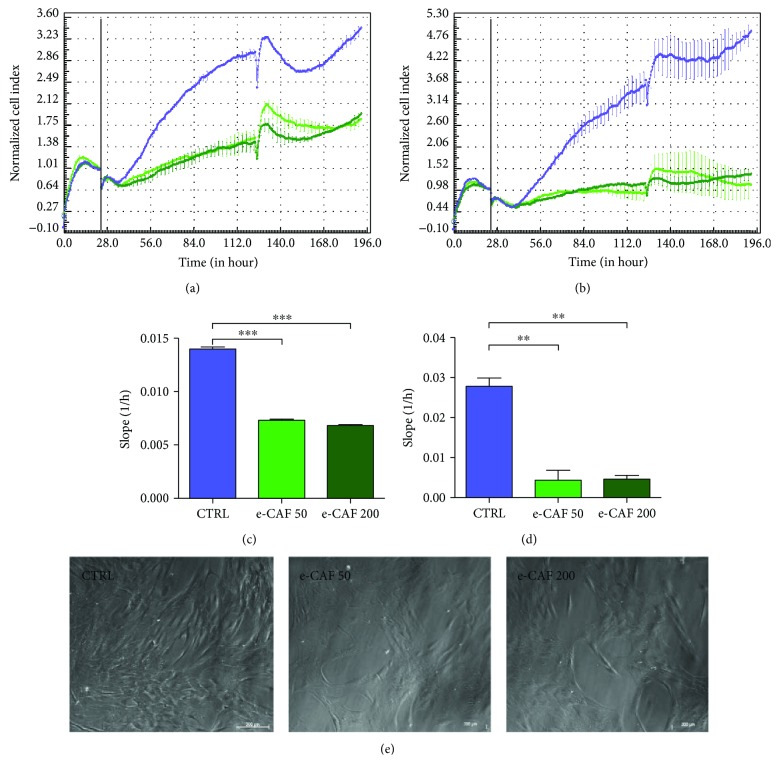
Effects of e-CAF on ADSC growth. RTCA graphs at two seeding densities, 2000 (a) and 1000 (b) cells/well. Normalization point was set at 24 h (vertical bar). Points represent the mean normalized cell index (CI) of three experiments run in triplicate ± SD for untreated ADSC (blue), cells treated with 50 *μ*g/mL e-CAF (light green), and cells treated with 200 *μ*g/mL e-CAF (dark green). Slope values computed from the RTCA between the normalization point (day 0) and the end of the experiment (day 7) at 2000 (c) and 1000 (d) cells/well seeding densities. ^∗∗^*p* ≤ 0.01 and ^∗∗∗^*p* ≤ 0.001. (e) Representative photographs of ADSC cultures from each group at day 7.

First, we investigated the effects of e-CAF on the growth of ADSC. RTCA experiments showed that the addition of e-CAF to culture media caused a significant reduction in the rate of growth of ADSC. This effect of e-CAF on ADSC cultures was observed at both concentrations (50 *μ*g/mL and 200 *μ*g/mL), as well as at two different densities of cell seeding. The significant inhibitory effect of e-CAF was patented as soon as 24 hours after the addition of the product to the cultures and remained for the following 7 days of the study (Figures 1(a)–1(d)). The disturbances in the curves observed at day 4 were caused by the medium change procedures. The analysis of the slopes corresponding to the interval of the experiment showed significant differences between the treated groups and control group, with greater values in the case of untreated ADSC curves.

Next, we investigated whether e-CAF had any effects on the morphology of ADSC. The presence of e-CAF in the culture media induced morphological changes in the cells at both concentrations. e-CAF-cultured cells presented bigger sizes and a more polyhedral shape at 7 days (Figure 1(e)). Changes in ADSC morphology were observed since day 5 (data not shown). Furthermore, at 7 days of culture, ADSC treated with e-CAF showed higher levels of cytoplasmic refringence and complexity, suggesting the presence of a more developed cytoskeleton. In contrast, ADSC cultivated in medium alone mainly showed a spindle-shaped, more numerous cells which covered more culture surface, near to confluence.

**Figure 2:**
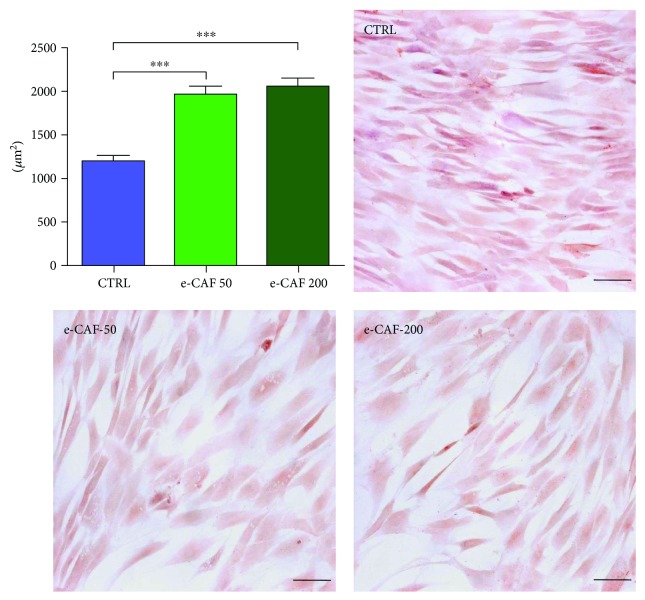
Effects of e-CAF on ADSC size and morphology. Morphometrical studies of the cells at culture day 7. Representative photographs of the study groups are shown (haematoxylin-eosin, bars: 100 *μ*m). Graph represents the mean area of cells in each group, measured within nine fields (10 cells/field) of three experiments. An increase of the cellular size in groups exposed to 50 *μ*g/mL e-CAF and 200 *μ*g/mL e-CAF can be observed. ^∗∗∗^*p* ≤ 0.001.

We also performed haematoxylin-eosin staining of the ADSC cultures in the presence or absence of e-CAF after 7 days of culture (Figure 2). There was no evidence of increased cell death in ADSC cultures due to the presence of e-CAF with any of the concentrations. The mean size of the cells, measured as cellular area, was higher in the ADSC treated with both concentrations of e-CAF than in the control group. Since the RTCA showed a higher growth of control cultures, and the cells of this group were markedly smaller, these results show a double effect of e-CAF upon ADSC, both reducing their proliferation and promoting the gain of cellular volume.

### 3.2. e-CAF Increases the Proportion of *α*SMA-Positive ADSC

**Figure 3:**
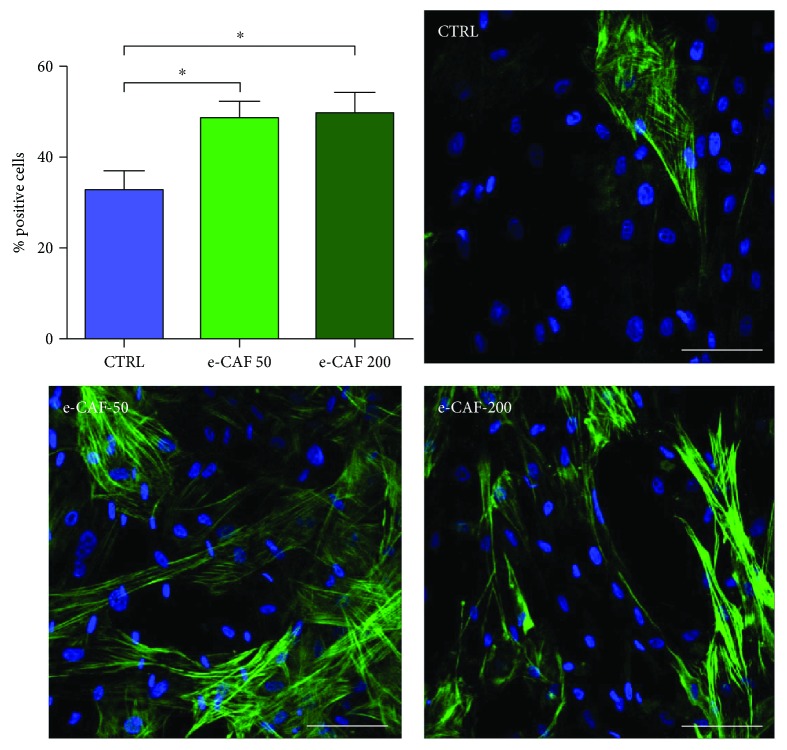
Effects of e-CAF on *α*SMA expression. Immunofluorescence detection of the myofibroblast marker *α*SMA (green) in the three groups (bars: 50 *μ*m). Cell counts (graph) showed a higher percentage of positive cells for this marker in both e-CAF-treated groups. The results are expressed as the mean of positive cells per field, measured within nine fields of three experiments. ^∗^*p* ≤ 0.05.

Next, we investigated the effects of e-CAF on the differentiation of ADSC to myofibroblasts by immunofluorescent detection of the expression of *α*SMA, a cytoskeletal protein commonly used as a myofibroblast marker (Figure 3). The quantification of the proportion of positive cells showed significant differences in the treated groups. Although positive cells were also found in the control group (32.76% ± 4.11%), higher values were observed in both e-CAF-50 (48.59% ± 3.62%) and e-CAF-200 (49.80% ± 4.41%) groups, without significant differences between them.

Furthermore, we also investigated the morphology of ADSC expressing *α*SMA. In both e-CAF-treated and e-CAF-untreated ADSC, *α*SMA-positive cells showed aligned cytoskeletal microfilaments. It was also observed that these cells tended to possess a greater extent of cytoplasm, frequently concurrent with polyhedral shapes.

### 3.3. e-CAF Upregulates the Expression of *α*SMA in ADSC

**Figure 4:**
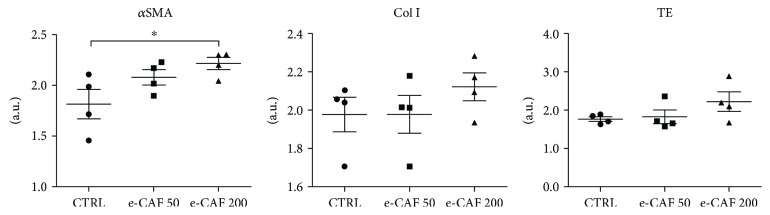
Effects of e-CAF on ADSC gene expression. Gene expression of *α*-smooth muscle actin (*α*SMA), collagen I (Col I), and tropoelastin (TE) in the three experimental groups, measured by RT-qPCR. GAPDH was used as reference gene. The results are log-transformed and are expressed as arbitrary units. A trend to an increase in the expression of the myofibroblast marker and the matrix proteins in e-CAF-treated groups was observed, with significant differences in *α*SMA expression between untreated and 200 *μ*g/mL e-CAF groups. ^∗^*p* ≤ 0.05.

To further assess the extension to which e-CAF induces ADSC differentiation, mRNA levels of myofibroblast markers, such as *α*SMA, type I collagen, and tropoelastin, were measured by real-time quantitative PCR in ADSC cultured in the presence and absence of e-CAF for 7 days (Figure 4). The expression of *α*SMA showed an increase in the treated groups compared to the control group, reaching statistical significance in the e-CAF-200 group. No significant differences were observed in the expression of the extracellular matrix proteins type I collagen and tropoelastin, although in both cases, the e-CAF-200 group showed a clear trend to express higher mean values of these proteins than the control group.

## 4. Discussion

Aging causes changes in the structure and physiology of all tissues and organs. However, being the outermost part of the organism, the skin is exposed to additional aggressions that aggravate this process, leading to the clinical condition of photoaging [8]. Extensive research has been carried out aiming to find suitable agents capable of overcoming these age-related effects on the skin. To this end, many natural products have been assessed as reparative agents, mainly due to their antioxidant and anti-inflammatory properties [30, 31]. In this work, an extract obtained from the eggs of the mollusk *Cryptomphalus aspersa* (e-CAF) has been tested on human ADSC cultures in order to assess its effects on this cell lineage which may give rise to cells with cutaneous phenotype.

The growth of the cultures exposed to e-CAF was measured by RTCA. This novel technology is based on the changes of impedance registered in a cell culture e-plate covered by electrodes, allowing a noninvasive and label-free monitoring of the cultures [32]. This system has proven to be a powerful tool in the evaluation of proliferation, migration, and invasion of different cell cultures [33, 34], in the measurement of cytotoxic effects of different substances [32, 35] and in the assessment of stem cell viability and optimal time of use for cell therapy [36]. In our study, two different concentrations of e-CAF caused a decrement of ADSC proliferation rate, without observing any cytotoxic effect. Instead, the monitoring of the replica plates showed morphological changes in the e-CAF groups compatible with a cell differentiation process compared to control cells, and these observations were also confirmed by quantification of cellular size. Since in the RTCA experiments, both cell proliferation and cell growth can contribute to an increase of the CI, the bigger sizes of treated cells indicate that the differences between the e-CAF and control groups in proliferation rates are even more robust than those recorded. These results are aligned with the finding of Alameda et al. [29] that observed a significant decrease in proliferation of human hair dermal papilla stem cells (HHDPCs) with similar concentration of e-CAF (50 and 200 *μ*g/mL).

The observed tendency to change the spindle-shaped characteristic of ADSC [15] to a more polyhedral one, with a prominent cytoskeleton, and the reduced proliferation rate suggested a possible effect of e-CAF favoring differentiation of ADSC towards myofibroblast lineage. Therefore, we examined the presence of *α*SMA, the most widely used marker to identify myofibroblasts [19, 37], in our cells. While positive cells were found in all groups, e-CAF treatment significantly increased the percentage of *α*SMA-positive cells compared to untreated cells, which confirmed our hypothesis. Accordingly, ADSC have been previously reported as capable to differentiate into *α*SMA-expressing cells, including vascular smooth muscle cells [38] and myofibroblasts, which also show high plasticity in the modulation of their contractile and migratory phenotypes [18]. This plasticity could partially explain the mild differences observed in collagen type I and tropoelastin mRNA levels, as the expression of ECM proteins is greatly upregulated in contractile myofibroblasts, induced mainly by TGF-*β*1 [18]. Additionally, these cultures lack many stimuli that are naturally present *in vivo* and cause ECM secretion by myofibroblasts, such as mechanical forces, and especially TGF-*β*1 and its interaction with many components of the matrix, which greatly modulates their action [37, 39, 40]. It must be noted, however, that the greatest level of expression for the three studied genes was found in the e-CAF-200 group, which could be suggestive of a more advanced differentiation in the group treated with the higher concentration. The observed effect of e-CAF favoring differentiation of ADSC towards myofibroblast lineage is not restricted to these stem cells. It has ben also shown that e-CAF exert differentiation effects of HHDPCs to the main skin cell lineages towards myofibroblast, a specific fibroblastoid phenotype [29]. The molecular mechanism of the myofibroblast differentiation promotion observed with e-CAF treatment has not been established, but it is possible to suggest it may be able to mimicry the TGF-*β*1 regulatory effects.

Aging has been related to a failure in fibroblast-to-myofibroblast differentiation, concurrent with lower generation of hyaluronic acid [41]. Being myofibroblasts as one of the main ECM-secreting cells, the inductive effect upon ADSC of e-CAF towards this lineage could be beneficial in the treatment of cutaneous aging. Related to this, recent studies have shown that e-CAF also has several proregenerative effects on other cutaneous cell types, such as keratinocytes and fibroblasts [28].

Moreover, functional stem cell units have been described throughout all layers of human skin: hair follicle bulge, interfollicular epidermis, dermal papillae, and perivascular hypodermal adipose tissue. Their secretome may be of great use due to the broad range of growth factors and other signalling molecules they produce [42]. However, in the course of aging, important changes occur in stem cell populations, reducing their pool, diminishing their functionality, and altering their niches [43–45], which in turn aggravate the aging process. Therefore, the existence of treatments capable of modulating stem cell activity and differentiation would be desirable.

In the last few years, ADSC have become a promising tool in the treatment of skin aging. Stem cell-based therapies offer tremendous potential for skin regeneration. ADSC have shown to paracrinally induce collagen synthesis and angiogenesis, while reducing the levels of matrix metalloproteinase-1 (MMP-1) and cell apoptosis [12, 13]. It has also been suggested that ADSC could play a role in preventing the accumulation of advanced glycation end-products (AGEs) associated with aging [14]. Since the activity of these stem cells can be so beneficial to ameliorate the effects of skin aging, resident ADSC from the perivascular hypodermis emerge as a potential target for antiaging treatments, such as e-CAF.

## 5. Conclusion

Our results show that e-CAF promotes ADSC differentiation to myofibroblasts and should be considered as a potential agent for the prevention and treatment of skin aging. These modulatory effects of e-CAF on ADSC expand those described on HHDPC differentiation and indicate that e-CAF could exert its activity on multiple types of skin cells and should be considered as a potential agent for the prevention and treatment of skin aging.
